# Mesoporous Silica Nanoparticles in Bioimaging

**DOI:** 10.3390/ma13173795

**Published:** 2020-08-27

**Authors:** Daohe Yuan, Connor M. Ellis, Jason J. Davis

**Affiliations:** Department of Chemistry, University of Oxford, South Parks Road, Oxford OX1 3QZ, UK; daohe.yuan@chem.ox.ac.uk (D.Y.); connor.ellis@chem.ox.ac.uk (C.M.E.)

**Keywords:** bioimaging, imaging modality, magnetic resonance imaging, optical imaging, multi-modality imaging, nanoparticles, mesoporous silica nanoparticles

## Abstract

A biomedical contrast agent serves to enhance the visualisation of a specific (potentially targeted) physiological region. In recent years, mesoporous silica nanoparticles (MSNs) have developed as a flexible imaging platform of tuneable size/morphology, abundant surface chemistry, biocompatibility and otherwise useful physiochemical properties. This review discusses MSN structural types and synthetic strategies, as well as methods for surface functionalisation. Recent applications in biomedical imaging are then discussed, with a specific emphasis on magnetic resonance and optical modes together with utility in multimodal imaging.

## 1. Introduction

Mesoporous silica materials were first reported in 1992 by the Mobil Research and Development Corporation, and named the Mobil Composition of Matter 41 (MCM-41) [1,2]. Since then, extensive efforts have been made in this field with numerous types of mesoporous silica nanomaterials successfully synthesised, differing in morphology, pore size, etc. In general, morphological control is achieved by varying the reaction conditions (e.g., pore directing surfactants, reaction precursors, reaction temperature, etc.; see Table 1). In recent years, mesoporous silica nanomaterials (MSNs) have been widely used for a wide range of applications such as those spanning drug delivery [3,4,5,6], diagnostics [7] and catalysis [8].

Non-invasive imaging techniques represent a powerful asset in disease diagnosis and, thus, of improved patient outlook [9,10]. The techniques available include magnetic resonance imaging (MRI), optical imaging (OI), positron emission tomography (PET), computed tomography (CT), single photon emission computed tomography (SPECT) and ultrasound (US). Through the use of an associated contrast agent, imaging clarity can be markedly improved and, moreover, specific physiological states/regions can be highlighted. In recent years, a broad range of nanoparticle-based contrast agents have been developed, many of which offer notable advantages over conventional molecular analogues. These include superparamagnetic iron oxide nanoparticles (SPIONs) for MRI contrast acquisition [10,11] and inorganic semiconducting nanoparticles, such as quantum dots (QDs) or upconverting nanoparticles (UCNPs) for OI. Among the nanomaterials available, those based on a mesoporous silica platform have particularly attractive physicochemical features such as tuneable shape/size/morphology, large surface area/pore volume, well-defined mesoporous structures, high colloidal/thermal stability, good biocompatibility and ease of surface functionalisation [12,13,14,15]. There exist, now, many examples of their applications as both effective basic imaging agents and in support of imaging-guided therapy, multimodal imaging, etc. [7,16,17,18].

This review covers the synthesis of MSNs with various structures, followed by an introduction to the strategies employed in their functionalisation. The most recent applications of MSN-based nanomaterials in bioimaging, including single-modal and multimodal techniques, are then discussed.

## 2. Synthesis 

### 2.1. Synthesis of Mesoporous Silica Nanoparticles by Sol–Gel Chemistry

Colloidally stable silica particles were first reported by Stöber et al. in 1968, where monodisperse micron-sized silica spheres (ranging from 0.05 µm to 2 µm) were formed by the co-condensation of tetraalkyl silicates using alcoholic ammonia as a catalyst [33]. Kresge et al. built on this foundation producing an ordered mesoporous silica material, where “mesoporous” refers to a porous material of pore size between 2 nm and 50 nm, denoted MCM-41 [1]. This discovery brought the birth of ordered mesoporous nanomaterials, with MCM-41 becoming the most worked. Five years later, a modification of the Stöber method was reported to produce sub-micron MCM-41 structured spheres through the introduction of an additional cationic surfactant, in a sol–gel technique. This is now the dominant method applied in the fabrication of MSNs [34,35].

Traditionally, a sol–gel process refers to a wet chemical reaction wherein small molecular monomers (sol) are transformed into an integrated solid network (gel). The preparation of MSNs by this method uses a silicon alkoxide as a sol material that undergoes a series of hydrolytic and condensing processes to form gel-like MSNs. These hydrolysis and condensation steps are summarised as:

$$\begin{array}{ll} \mathrm{Hydrolysis}: & \mathrm{Si}-{\left(\mathrm{OR}\right)}_{4}+H_{2}O\to {\left(\mathrm{OR}\right)}_{3}-\mathrm{Si}-\mathrm{OH}+\mathrm{ROH}\\ \mathrm{Alcohol}\;\mathrm{condensation}: & \mathrm{Si}-{\left(\mathrm{OR}\right)}_{4}+\mathrm{HO}-\mathrm{Si}-{\left(\mathrm{OR}\right)}_{3}\to {\left(\mathrm{OR}\right)}_{3}-\mathrm{Si}-O-\mathrm{Si}-{\left(\mathrm{OR}\right)}_{3}+\mathrm{ROH}\\ \mathrm{Water}\;\mathrm{condensation}: & {\left(\mathrm{OR}\right)}_{3}-\mathrm{Si}-\mathrm{OH}+\mathrm{HO}-\mathrm{Si}-{\left(\mathrm{OR}\right)}_{3}\to {\left(\mathrm{OR}\right)}_{3}-\mathrm{Si}-O-\mathrm{Si}-{\left(\mathrm{OR}\right)}_{3}+H_{2}O \end{array}$$

where R represents an alkyl group such as methyl, ethyl, etc. It is worth mentioning that the slowest hydrolysis rate occurs at pH 7.0, so the reaction is usually conducted under either acidic- or basic- catalysed conditions [8,36]. 

**Figure 1:**
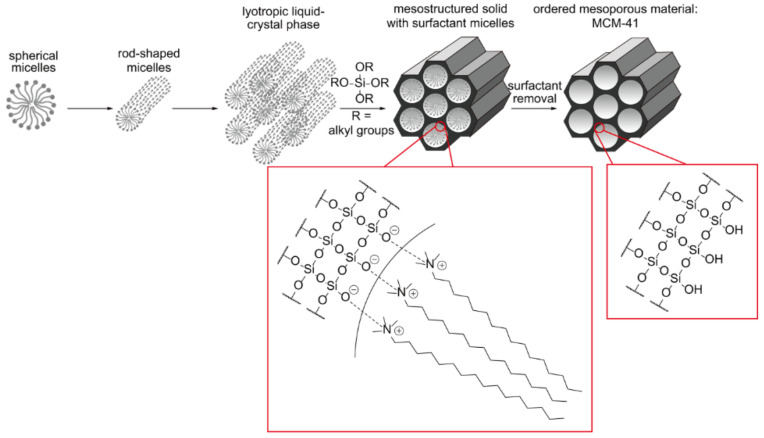
A schematic illustration of the synthesis of MSNs. Initially, surfactant micelles are formed which then self-assemble forming a lyotropic liquid-crystal phase. Condensation of the silica source around the surfactant template and subsequent surfactant template removal produces MCM-41. Here, tetraethyl orthosilicate (TEOS) is used as the silica source and cetyltrimethylammonium bromide (CTAB) as the surfactant template.

The sol–gel chemistry behind an MSN formation is complex, relying on the use of surfactant templates to introduce porosity. A typical scheme for the synthesis of MCM-41 is shown in Figure 1.

The first step in the mechanism is the formation of surfactant micelles in an aqueous solution. The individual micelles self-assemble, forming a rod-like structure and align to form a lyotropic liquid crystalline phase. Introduction of the silica source (e.g., tetraethyl orthosilicate) begins the construction of the silica architecture around the surfactant cylinders, with the final micelle core/silica shell fully formed after ~400 s [37]. The surfactant template is subsequently removed, usually by an acidic extraction or calcination method, leaving MCM-41.

There are reported works investigating reaction kinetics, which have highlighted a number of general trends relevant to control the structure and morphology. One important factor is the choice of surfactant. The packing parameter g for surfactant molecules defines the assembled micelle shape and, thus, the morphology of the meso-product. This is given by, Equation (1):

(1) g=V/al

where V is the volume of the hydrophobic surfactant chain, a the effective head group area and l the surfactant chain length. From highly-curved to flat lamellar morphologies, g increases in the order L_1_ (spherical micelle, $g\;<\;\frac{1}{3}$) < H_1_ (hexagonal, $\frac{1}{3}<g<\frac{1}{2}$) < V_1_ (cubic, $\frac{2}{3}<g<\frac{3}{4}$) < L_α_ (lamellar, $\frac{1}{2}<g<1$) [38]. By choosing a surfactant g value it is possible to synthesise MSNs with a specific morphology (e.g., H_1_ gives the MCM-41 structure, L_α_ gives MCM-48 structure). In addition, the pH of the reaction mixture, and, hence, silane/surfactant ionisation states, is important for control over structure and morphology with this closely related to the type of interaction between the surfactant headgroup and silicates. For example, when the pH is below the isoelectric point of silica (pH ≈ 2.0), weak electrostatic forces are present on the interface of the surfactant–silicate assemblies (S^+^X^−^I^+^, where S^+^ corresponds to the cationic surfactant, I^+^ is the positively charged silicate (–SiOH_2_^+^) and X^−^ is a counter anion e.g., halides or nitrate). The interaction in these organic–inorganic assemblies is different from that of its corresponding alkaline analogue (S^+^I^−^, where S^+^ is the cationic surfactant, I^−^ is the negatively charged silicate (–SiO^−^)). In general, acidic synthesis strategies produce MSNs with diverse morphologies [39], including fibres, spheres, ropes, doughnuts, etc. [27]. By comparison, the fabrication of MSNs under alkaline conditions often yields small sub-micrometre-sized nanoparticulates due, in large part, to a faster nucleation rate [40].

In addition to general morphological control, other structural features (such as mesopore size, particle size, shape, etc.) are reaction-condition-dependent in a manner that is broadly controllable and has been recently summarised [36,39].

### 2.2. Synthesis of Hollow MSNs and Core–Shell Structured MSNs

There has been, over the past decade or so, an ever-increasing demand for multifunctional mesoporous silica nanoparticles of specially designed structures and functions. Hollow mesoporous silica nanoparticles (HMSNs) and core–shell structured MSNs have been particularly high-profile examples of this. 

**Figure 2:**
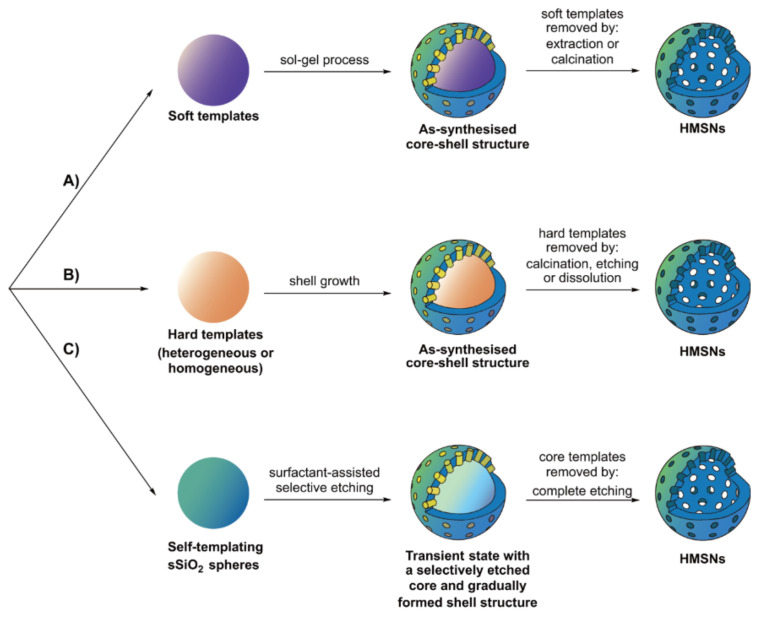
A schematic detailing the three synthetic routes for fabrication of hollow mesoporous silica nanoparticles (HMSNs), namely “soft-templating”, “hard-templating” and “self-templating”. All three routes start from the formation of the core template. Route (**A**) is a two-step process, resulting in the formation of HMSNs after the removal of both the mesopore and core templates. Route (**B**) illustrates the formation of HMSNs by a hard-templating method after the removal of both the mesopore and core templates. Route (**C**) shows the process of self-templating fabrication of HMSNs. The final hollow mesoporous silica nanoparticles are obtained by a complete etching of self-templating solid silica spheres (sSiO_2_). Common etching agents include strong acids such as HCl and HF, an alkaline medium such as ammonia and NaOH, NaBH_4_ or hot water [49].

The former refers to MSNs composed of an interior void space with penetrating channels integrated into the silica shell. There are some important features of HMSNs: (i) the interstitial hollow space is able to act as a reservoir, (ii) guest molecules are free to diffuse in/out of the silica shell through the pore channels and (iii) it is easy to chemically modify the exterior silica surface (via abundant silanol groups). These features mean HMSNs represent ideal candidates for both the loading and delivery of chemical agents (e.g., carbon dots, organic dyes, fluorine compounds, etc.) [41,42,43]. HMSNs can be produced by several different methods—“soft-templating”, “hard-templating” and “self-templating” [44,45]—as illustrated by Figure 2.

The main difference across these is the strategy employed for the generation of the interior cavity. A soft-templating method uses “soft” templates such as micelles, microemulsion droplets, vesicles and gas bubbles, while the hard-templating method is based on a rigid solid template such as a polymer latex or inorganic nanoparticle. The self-templating method often utilises solid silica spheres (sSiO_2_) produced by the Stöber process as the core template. For hard-templating, three steps are required for the formation of HMSNs: (i) the formation of a nanosized template, (ii) the construction of the mesoporous silica shell on the core and (iii) the removal of the template to generate the integrated cavity [46]. The soft-templating approach for HMSN formation also requires three steps: (i) the fabrication of a core template with a simultaneous nucleation of precursors/surfactants, (ii) the construction of the mesoporous structure around the soft template and (iii) the removal of templates/surfactants by calcination or extraction. Self-templating is a modification of the hard-templating method, using sol–gel derived silica as the core template, which can then be directly converted into HMSNs [47]. In general, the mechanism is better described as a two-step process: (i) formation of the core template and (ii) transformation of the template material into a hollow mesoporous shell. It is worth mentioning that self-templating is much more recent in its conception [48]. In a typical self-templating reaction, an alkaline cationic surfactant-assisted strategy (Figure 2C) has been shown to facilitate the selective etching of the core interiors while keeping the surface intact. This arises from the strong electrostatic interactions between the surfactant and the silica surface under the alkaline conditions of the reaction, leading to the formation of hollow structures from a non-porous core template [47]. Although soft-templating and self-templating strategies represent facile preparatory methods under mild reaction conditions, hard-templating has become more prevalent in recent years [45] due to the apparent greater control it brings over key nanoparticulate features (such as monodispersity, size dimension, shell thickness, mesoporosity, etc.).

**Figure 3:**
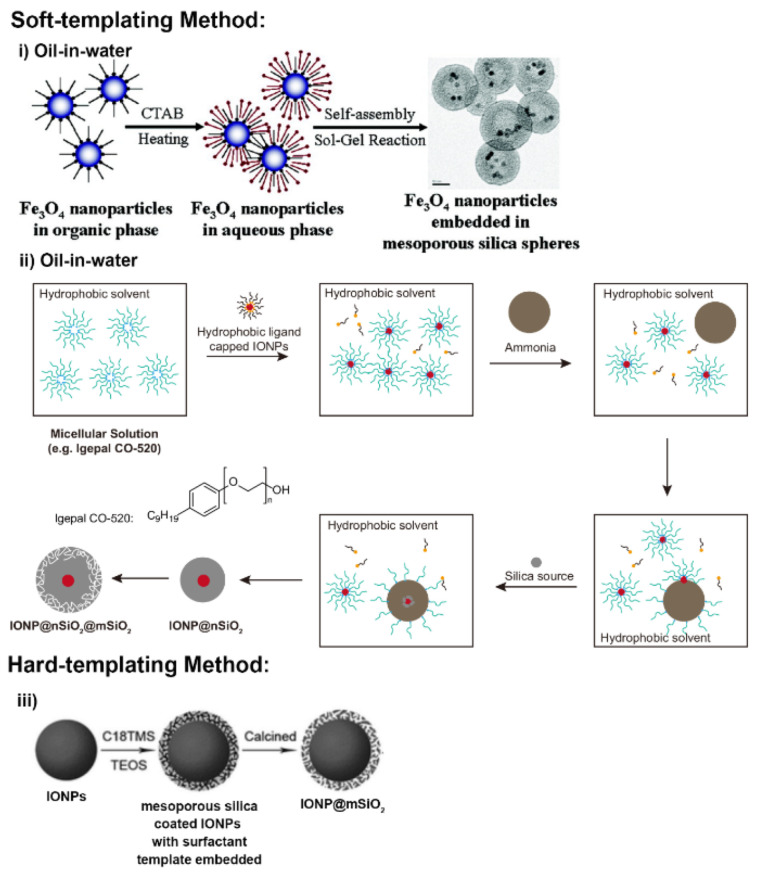
A schematic illustration of the synthesis of magnetic mesoporous silica nanoparticles by different methods. (**i**) Synthesis using an oil-in-water technique. Hydrophobic-ligand-capped iron oxide nanoparticles dispersed in a nonpolar organic solvent are added to an aqueous solution containing cetyltrimethylammonium bromide (CTAB) and TEOS, allowing the formation of core–shell magnetic mesoporous silica nanoparticles (M-MSNs). Adapted with permission from reference [51]. Copyright (2006) American Chemistry Society. (**ii**) Synthesis using a water-in-oil method. The synthesis starts with micellular solution of an amphiphilic surfactant (such as Igepal CO-520), and core–shell iron oxide nanoparticle@nSiO_2_ (IONP@nSiO_2_) with a reverse-microemulsion system formed on the addition of ammonia. Finally, the core–shell M-MSNs are produced by the introduction of an additional mesoporous silica shell (mSiO_2_). (**iii**) Synthesis using a hard-templating strategy. This method starts with the introduction of mesoporous silica structures on the surface of native iron oxide nanoparticles by adding the silica source and the pore directing agent (e.g., TEOS and cetyltrimethylammonium salt). The desired M-MSNs are produced after calcination to remove the embedded surfactant template. Adapted with permission from reference [54]. Copyright (2009) The Royal Society of Chemistry.

Core–shell MSNs are characterised by the nanostructure having an integrated functional nanocore (e.g., SPIONs, Au NPs, UCNPs, etc.) within a mesoporous silica shell. Magnetic mesoporous silica nanoparticles (M-MSNs) constitute a particularly dominant class of these. Soft or hard templating synthesis methods are applied here (Figure 3).

For the former, these are water-in-oil (W/O) and oil-in-water (O/W) [50,51] with a pore directing agent (PDA) such as cetyltrimethylammonium bromide (CTAB) employed, enabling the phase transfer of oleic acid-capped iron oxide nanoparticles (IONPs) from the hydrophobic solvent into the aqueous solvent. A subsequent sol–gel reaction of tetraethyl orthosilicate (TEOS) with PDA-stabilised magnetic nanocrystal produces the mesoporous silica shell, with the desired M-MSNs obtained after the removal of the PDA. However, in a typical O/W (also known as reverse microemulsion) procedure (Figure 3ii), M-MSNs are fabricated through a more complicated process that involves both the formation of IONP@sSiO_2_ and a subsequent introduction of mesoporous silica outer shell. The first step is the formation of a micellular structure of the functional amphiphile (e.g., Igepal CO–520) in oil. Then, the hydrophobic ligand coated IONPs are added into the emulsion and a ligand exchange occurs between the ligands present on the IONP surface and the amphiphiles in solution. After the addition of an aqueous solution containing the silica source (in some cases, ammonia is additionally added) to the organic phase, a reverse microemulsion forms the amphiphilic surfactant encapsulated IONPs. The addition of TEOS results in the generation of IONP core-homogenous silica shell nanoparticles through condensation of the hydrolysed silica sol on the IONP surface [52]. The final step uses an additional amount of TEOS and PDA to generate the silica shell with mesoporosity achieved on the removal of the PDA, thus generating M-MSNs [53]. A hard-templating method can also be used to fabricate M-MSNs (Figure 3iii), where a functional hard template (such as native hematite nanoparticles) is directly mixed with a silica source (TEOS) in a one-pot synthesis. After removal of the surfactant template (e.g., by calcination), the core–shell structured M-MSNs are obtained [54].

### 2.3. Functionalisation of Mesoporous Silica Nanoparticles

A great advantage of MSNs is the ability to introduce a desired functional group to a specific region within the particle, making the platform immensely versatile with wide ranging applications e.g., diagnostic imaging and sensing [55,56]. This chemical modification can be achieved by two main methods: post synthetic functionalisation and co-condensation. 

Post synthetic functionalisation (also known as post-grafting) refers to the immobilisation of additional chemical moieties on the particulate surface after forming the mesoporous silica nanostructure. The chemistry underpinning this method involves utilising abundant silanol groups present upon the particle surface, readily modified by chlorosilanes or alkoxysilanes [57]. Much work has now reported on the introduction of functional silanes (e.g., vinyl-[58], cyanoethyl-[59], aminopropyl-[59,60], chloro-[61] (trimethoxy)silane) by this method. Post synthetic functionalisation can be performed either prior to the removal of the surfactant template (known as pore-protection grafting) or after the surfactant extraction. The former methodology gives exclusive external modification of MSNs [62], whereas the latter may generate both [63]. However, for surfactant-extracted MSNs, it is still possible to exclusively immobilise organic moieties upon the exterior surface by a post synthetic approach albeit with greater synthetic effort (diffusion-control grafting) [64,65].

Co-condensation is another method to introduce additional functionalities (such as those associated with nanoparticles, organic functional groups and/or metal complexes), in the presence of a structure-directing agent (SDA) [63]. In comparison with post synthetic functionalisation, the co-condensation approach offers a more homogenous distribution of the introduced functionality within the pore channels. This is because the functional moieties are direct components of the silica matrix, ensuring good accessibility of the introduced moiety throughout the pores. In addition, other issues that are prevalent in post-grafting (such as the blocking of pore channels and limited pore area/volume) do not occur in this approach. However, there are some drawbacks, namely: (i) the degree of mesoscopic order can be perturbed if the concentration of the functional moieties is very high [66] and (ii) the density of functionality typically incorporated by this technique is lower [66]. 

**Figure 4:**
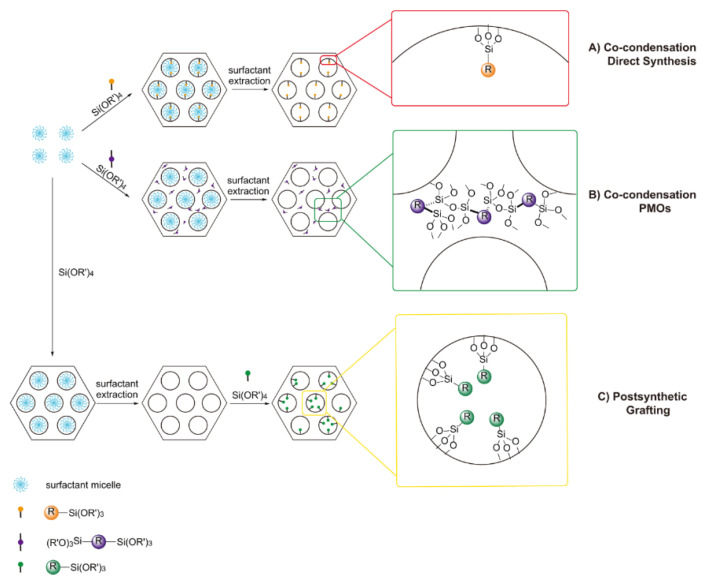
The immobilisation of chemical functionalities by both co-condensation and post synthetic functionalisation. Path (**A**) shows the direct synthetic co-condensation functionalisation by organo-substituted trialkoxysilanes on the surface of silica mesopores. Path (**B**) indicates the co-condensation process for periodic mesoporous organosilicas (PMOs), where bridged organosilanes (R’O)_3_Si-R-Si(OR’)_3_ and tetraalkoxysilanes are within the three-dimensional network structure of the silica matrix. Path (**C**) illustrates the post synthetic grafting of terminal trialkoxyorgnanosilanes.

There are two nanoparticulate platforms that can be produced by the co-condensation method: direct synthetic (also called one-pot) organically functionalised MSNs and periodic mesoporous organosilicas (PMOs). One-pot synthesis usually involves the use of terminal trialkoxyorganosilanes R-Si(OR’)_3_, tetraalkyl orthosilicates Si(OR’)_4_ with an SDA (e.g., cetyltrimethylammonium bromide). On the other hand, if R-Si(OR’)_3_ is substituted by the bridged organosilane precursor (OR’)_3_-Si-(OR’)_3_, homogenous hybrid organic–inorganic MSNs, where organic units are covalently incorporated within the three-dimensional silica matrix (more specifically, homogenously distributed in the pore walls), are produced. This material is known as periodic mesoporous organosilica (PMO) and exhibits some advantages compared to conventional MSNs, namely a high loading capacity and a uniform distribution of organic groups within the framework [67]. PMOs have shown a great potential in many applications such as optics [68], catalysis [69] and adsorption studies [70]. Figure 4 is a schematic summary of the post synthetic grafting process as well as both co-condensation methodologies. 

## 3. Magnetic Resonance Imaging

Magnetic resonance imaging (MRI) is a powerful imaging modality capable of identifying a wide variety of disease states due to its convenience, penetration depth and innate three-dimensional resolution. The intrinsic low sensitivity is overcome through the use of a contrast agent [71], classified as being *T*_1_ or *T*_2_ active, depending on the generated contrast enhancement (longitudinal or transverse relaxation respectively) [72]. *T*_1_ active contrast agents brighten the region of interest (positive contrast) with *T*_2_ active contrast agents darkening the desired area (negative contrast) [73]. 

**Figure 5:**
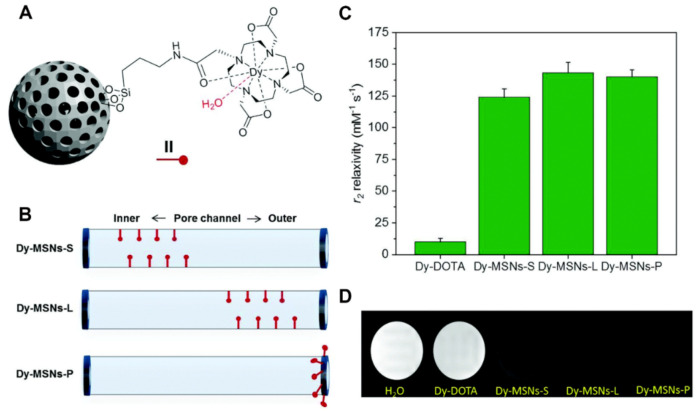
A figure detailing how the position of paramagnetic chelates within MSNs affects the relaxivity (*r*_2_) values. (**A**) General schematic of Dy-MSNs. (**B**) The different available locations for the tethered Dy-chelate. S, L and P refer to the synthetic procedure i.e., short-delay, long-delay and post-grafting, respectively. (**C**) Associated relaxivity values where the highest relaxivity can be observed for Dy-MSNs-L with *r*_2_ = 143.5 ± 8.2 mM^−1^ s^−1^ at 11.7 T. All three locations show substantially improved *r*_2_ in comparison to native molecular Dy-DOTA. (**D**) MRI phantoms characterising the response of the system. Adapted with permission from reference [80]. Copyright (2018) The Royal Society of Chemistry.

MSNs capable of shortening longitudinal relaxation rates can be produced by simply incorporating a paramagnetic ion within the silica architecture. Seminal work by Taylor et al. used this strategy to functionalise the MCM-41 material with Gd-Si-DTTA through siloxane linkers integrated within the silica nanoparticles [74]. This produced the first example of MRI-active MSNs with *r*_1_ = 28.8 mM^−1^ s^−1^ at 3 T, a scaffold that has since been adopted and modified by many groups, with *T*_1_ relaxivities of up to 33.57 ± 1.29 mM^−1^ s^−1^ at 7 T recorded for this platform. Our understanding of the operational principles has led to an appreciation of the importance of the location of the paramagnetic chelate in image contrast [75]. Magnetic resonance (MR) contrast generation can also be provided by alternative means through the integration of nanoparticles within the MSN architecture. This includes synthesis of MSNs with a core/shell architecture where the core is magnetically active e.g., SPION/MSN core/shell systems [76]. Alternatively, nanocomposites such as metal oxides can be confined to the pore channel of the MSN, with this reported by Ni et al. where gadolinium oxide loaded within the pores displayed effective *T*_1_ contrast capabilities [77]. While Gd^3+^ and Mn^2+^ promote positive contrast within the MRI scan, *T*_2_-active MSNs can be produced through the introduction of paramagnetic ions with anisotropic ground states, exhibiting short electronic relaxation times such as Dy^3+^ [78,79]. This has been recently reported by our group where Dy-DOTA complexes were covalently coupled through amino anchor points to generate Dy-MSNs with an effective transverse relaxation rate *r*_2_ = 143.5 ± 8.2 mM^−1^ s^−1^ at 11.7 T when Dy-DOTA resided in the outer pore channel of the particles (Figure 5) [80].

Since one of the inherent advantages of MSNs is the facile chemical tunability of the system, the examples included in this review focus on MSNs with an additional functional property. These include active targeting of a particular pathological region, therapeutic action and environmental responsivity.

### 3.1. Paramagnetically Doped MSNs

#### 3.1.1. Targeting MSNs

Particles smaller than 7 nm are rapidly excreted through the kidney glomerulus preventing long term toxicity issues and <100 nm is desirable to avoid uptake by the reticuloendothelial system [81]. Nanoparticulate systems possess, in general, an ability to passively target tumour cells through the enhanced permeability and retention (EPR) effect. Specifically, the poor associated lymphatic drainage and leaky vasculature of cancerous tissue can result in the accumulation of nanoparticles within the cells and allows imaging of the pathological region [82]. The inherent chemical tunability of the MSN architecture provides the possibility for additional active targeting to supplement this effect and improve contrast specificity. This has been reported, for example, by Hu et al. where surface tethering of MSNs with both Gd-DOTA and the cyclic arginine-glycine-aspartic peptide (cRGD) produced a system capable of MRI contrast enhancement, with *r*_1_ = 37.4 mM^−1^ s^−1^ at 0.5 T, and active targeting of α_V_β_3_ integrin receptors [83]. Alternative vectors include the use of MSNs functionalised with hyaluronic acid for lymph system targeting [84]. For these particles, gadolinium was introduced as part of the co-assembly process, with the doped MSNs exhibiting *r*_1_ = 11.2 mM^−1^ s^−1^ at 3 T. Another common targeting ligand is folic acid (FA), where functionalisation of MSNs has allowed imaging of cancer cells where the folate receptor is often overexpressed. FA functionalised, Gd-doped MSNs have been reported by Chan et al. with an associated brightening of the MRI scan in vivo [85]. Interestingly, the conjugation of MSNs with the Ri7 antibody through poly(ethylene glycol) (PEG) linkers allows for active targeting of micro vessel endothelial cells (BMECs) in the brain, allowing MSNs to report on a wide range of central nervous system diseases (e.g., Alzheimer’s and Parkinson’s) by virtue of an apparent ability to cross the blood–brain barrier (BBB) [86]. These particles contained Gd-DTPA chelates on the MSNs surface for MRI reporting with associated *r*_1_ = 21.9 mM^−1^ s^−1^ at 1 T. Such active targeting capabilities are often combined with the ability to respond to a particular biological stimulus so that imaging/therapy may be localised to specific pathological conditions. This forms the basis of the next section.

#### 3.1.2. Environmentally Responsive MSNs

The ability to respond to an environmental stimulus provides enriched diagnostic (and theragnostic) capabilities, with numerous applications including pH-responsive drug delivery and responsive MRI reporting [87]. Generally, MSN pore accessibility to the solution is manipulated to achieve stimuli-responsivity, with therapeutics released under specific environmental conditions or paramagnetic water access tuned. In work by Chen et al., for example, gadolinium doped upconverting nanoparticles (UCNPs) were tethered to the MSN surface by pH-sensitive acetal groups, acting as a pore accessibility gate keeper [88]. The particles were loaded with doxorubicin (DOX) which was released under an acidic microenvironment (such as that provided by the internal environment of tumour cells), through an acetal hydrolysis. In addition to pH-responsive drug delivery, Gd^3+^ containing UCNPs were shown to generate good positive MRI contrast, *r*_1_ = 6.9 ± 0.1 mM^−1^ s^−1^ at 1.5 T. In other work, MSNs have been functionalised with dopamine and poly(ethylene glycol) (PEG) to incorporate the prodrug banoxantrone (AQ4N) through acid-labile Fe^3+^-mediated coordination to dopamine [89]. AQ4N is a drug with increased efficacy in a hypoxic environment. As such, the tumour site was irradiated with a laser (670 nm) to induce increased tumour hypoxia through hyperthermia. Mn^2+^ was incorporated through coordination of the phenolic hydroxyl groups in dopamine enabling one to monitor the accumulation of the nanoparticles at the tumour site through positive contrast MRI. pH-responsive drug delivery has also been achieved by coating MRI-active MSNs loaded with Gd_2_O_3_ and DOX with a layer by layer assembly of positive and negatively charged polymers [90]. At a low pH, charge reversal results in the disintegration of the polymer network, releasing the anticancer drug DOX. The increased water accessibility to the MSNs’ pore channels also resulted in an increase in MRI signal intensity at pH 5.0. The particles were functionalised with folic acid for enhanced uptake at the tumour site. A manipulation of diffusive water access to an integrated paramagnetic site has also been reported by Pellico et al. where MSNs were internally doped with Gd-DOTA and the pore channel capped with the pH-responsive polymer poly(acrylic acid) (PAA) [91]. The conformational change of PAA from globular to extended polymer chains at elevated pH is accompanied by a fully reversible 130% increase in *T*_1_ relaxivity. An alternative report has used the irreversible capping of MSNs with MnO nanoparticles to achieve improved *r*_1_ at low pH [92]. Under acidic conditions, the MnO nanoparticles disassemble, releasing paramagnetic Mn^2+^ ions, with an increase in *r*_1_ from *r_1_* = 0.7 mM^−1^ s^−1^ at pH = 7.4 to *r_1_* = 5.6 mM^−1^ s^−1^ at pH = 4.5. 

**Figure 6:**
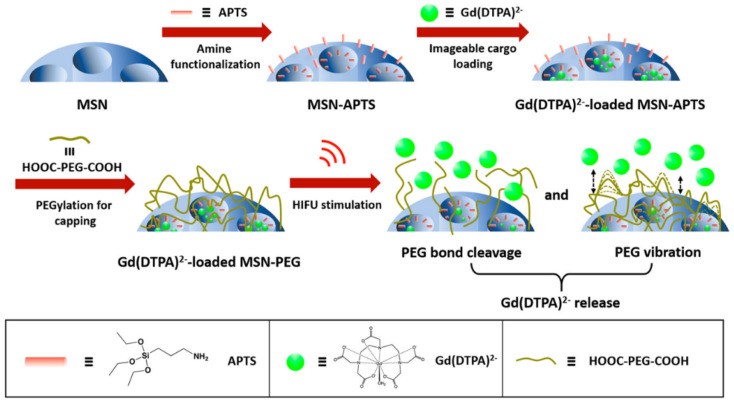
A schematic representation of high-intensity focused ultrasound (HIFU)-responsive magnetic resonance imaging (MRI)-active MSNs. In this work, the particles were initially amine Figure 2. (green) and the pore channels capped with poly(ethylene glycol) (PEG) (brown). Finally, the effect of HIFU stimulation on the release of Gd(DTPA)^2−^ can be observed. Reprinted with permission from reference [94]. Copyright (2019) American Chemical Society.

In addition to pH responsivity, there are reported works invoking different biological stimuli such as protein recognition of MSNs, with work by Huang et al. reporting a reversible switch in image contrast in the presence of biotin [93]. Streptavidin, which was used to cap the MSN pores and limit diffusive water access to integrated Gd-chelates, is displaced through strong streptavidin–biotin recognition improving both water accessibility and, thus, *T*_1_ response. Alternatively, the stimulus can be externally generated, as shown by the reported release of Gd(DTPA) from PEGylated MSNs by high-intensity focused ultrasound (HIFU, Figure 6) [94]. In this work, HIFU-generated mechanical forces were deemed responsible for the de-capping of the MSN pore channels, irreversibly releasing Gd(DTPA) and increasing *T*_1_ weighted image intensity.

### 3.2. Core–Shell Hybrid Nanoparticles

The entrapment of superparamagnetic iron oxide nanoparticles (SPIONs) within a mesoporous silica (MS) shell produces a highly functional and chemically tuneable MRI active system, with SPIONs able to provide either *T*_1_ or *T*_2_ contrast when encapsulated. This, of course, requires some consideration at the point of synthesis since the generated MRI contrast is inherently shell dependent. Usually, the SPION core is *T*_2_ active arising from a high saturation magnetisation value, promoting negative contrast within an MRI scan [73,95]. There is also the possibility of producing *T*_1_ active core/shell SPION/MSN nanocomposites if the SPION core is sufficiently small (less than 3 nm) as canting of magnetic spins result in paramagnetic-like behaviour [96]. This is exemplified in work by Hurley et al. where SPIONs with positive contrast capabilities formed the core of the SPION/MSN nanocomposite [97]. The porous nature of the silica shell allows facile diffusive water access to the SPION core with an associated *r*_1_ = 6.9 mM^−1^ s^−1^ at 1.4 T. Anti-cancer therapeutics, such as doxorubicin (DOX), can be loaded into the core/shell system for simultaneous imaging and drug delivery. In work by Chen et al., a selective etching of a middle silica layer using ammonium produced a cavity within the SPION/MSN core/shell structure, with an enhanced surface area for drug loading [98]. The electrostatic loading of DOX within the mesopores and cavity of the nanoparticles is reversed through protonation under acidic conditions facilitating a pH-responsive drug delivery within a tumour microenvironment (in addition to the negative contrast generation from the SPION core). To obtain greater control over DOX release, a responsive gatekeeper can be incorporated [87]. A recent example by Ménard et al. coated core/shell SPION/MSN particles with human serum albumin (HSA) through *iso*butyramide linkers for environmentally responsive drug delivery [99]. In the presence of protease enzymes, this HSA coating is degraded allowing for the enzyme-sensitive release of DOX to the surrounding medium. The SPION core provided *T*_2_ activity with *r*_2_ = 70.0 mM^−1^ s^−1^ at 1.4 T.

Alternative therapeutic function can also be integrated to complement MSN MRI diagnostic capability, as, for example, reported by Wang et al. where suicide gene therapy was mediated through a SPION/MSN platform [100]. Suicide gene therapy is the conversion of a non-toxic prodrug into a toxic metabolite through the introduction of a gene to promote apoptosis of cancerous cells [101]. For this aim, the herpes simplex virus thymidine kinase/ganciclovir (HSV-TK/GCV) system was used where the nanoparticles were loaded with GCV and subsequently coated with poly(ethylene glycol)-*g*-poly(_L_-lysine) (PEG-*g*-PLL) for electrostatic thymidine kinase (TK) plasmid adsorption. The particles exhibited *T*_2_ contrast capabilities with a corresponding *r*_2_ = 107.5 mM^−1^ s^−1^ at 3 T in addition to enabling the delivery of a suicide gene (TK) and prodrug (GCV) within hepatocellular carcinoma (HCC) cells. An alternative theragnostic system tethered folic acid (FA) to the silica shell enabling folate receptor targeting, (as noted) commonly overexpressed on cancerous cells such as those in breast cancer [102]. The MSNs were additionally loaded with DOX [103]. SPION/MSN composites have also been functionalised with tumour-targeting peptides with Xu et al. tethering VHPKQHR (Val-His-Pro-Lys-Glu-His-Arg) to the MSN shell for targeting of vascular cell adhesion molecule-1 (VCAM-1), providing direct negative contrast MRI reporting on atherosclerotic plaque in vivo [104].

**Figure 7:**
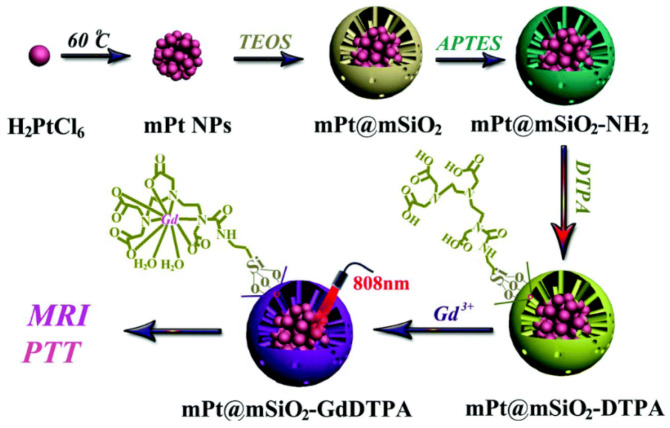
A core/shell hybrid based on a platinum core and MSN shell. Tethering with Gd-DTPA provides MRI activity with photothermal therapy mediated by the Pt core. APTES refers to (3-aminopropyl)triethoxysilane. Reprinted with permission from reference [111]. Copyright (2017) The Royal Society of Chemistry.

The use of an alternative core material can also be implemented; in one example, the SPION/MSN core has been modified in generating a manganese ferrite (MnFe_2_O_4_) analogue of enhanced magnetisation and, thus, contrast [105]. Alternatively, the use of a *T*_1_ active core provides positive contrast capabilities, with this reported for MSNs containing an internal gadolinium core [106]. These particles were additionally loaded with DOX and the photothermal agent indocyanine green in potentially enabling disease treatment through both chemo- and photo-means. The use of a pure manganese oxide core can also produce core/shell systems capable of enhancing generated positive contrast [107]. Such a construct has been functionalised with a prostate-specific membrane antigen for the *T*_2_ imaging of prostate cancer while additionally supporting *T*_1_ imaging through the manganese oxide core [108]. Additional therapeutic capacities can be integrated through the use of a photothermal active core; e.g., work by Gao et al. used a CuS core as a photothermal agent for photothermal therapy against HeLa cells, with the whole system PEGylated to improve both biocompatibility and blood circulation time [109]. Zhang et al. have also used a CuS core encapsulated in an MSN shell to provide the nanocomposite with a phototherapy capacity through near infrared (NIR) irradiation [110]. The MSN architecture also contained disulphide linkages responsive to glutathione (GSH) expression, exposing the CuS core and releasing the incorporated therapeutic agent DOX (enabling GSH-responsive drug delivery). In this example, the pore channels were capped with MnO_2_ nanoparticles, decomposing under the acidic tumour environment releasing the MRI-active paramagnetic Mn^2+^ ion enhancing the generated positive contrast. In addition to improved longitudinal monitoring through Mn^2+^ release, the Mn^2+^ was also shown to react with H_2_O_2_ present in tumour cells to produce OH radicals promoting cell apoptosis. Platinum core MSN particles exhibit the potential for photothermal therapy (PTT), mediated through the Pt core, on irradiation with an 808 nm laser (Figure 7) [111]. In one example of this, the MSN shell was additionally doped with Gd-DTPA in enabling MRI contrast.

The use of MSNs as contrast agents for MRI is, then, an extensive field with numerous reported works. As can be seen from the above examples the versatility is substantial with generated species capable of environmental responsivity, drug delivery and active targeting in addition to a potential longitudinal/transversal monitoring of disease. Both native MSNs and core/shell analogues have been applied with generated MRI contrast provided either by tethered paramagnetic chelates within the silica matrix, or through an active core. In some cases, this core can provide additionally therapeutic potential such as by photothermal means.

## 4. Optical Imaging

**Figure 8:**
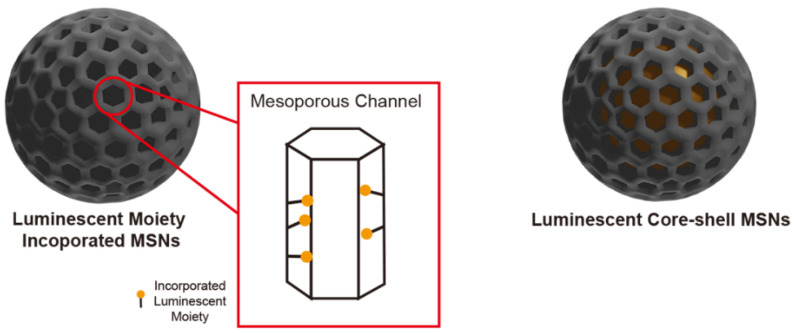
Schematic illustration of two types of MSNs as applied in optical imaging where a luminescent moiety is incorporated into the MSNs substructure (Section 4.1) or is confined within an MSN-wrapped luminescent core (Section 4.2).

Optical imaging (OI) is a non-invasive technique of high spatial resolution and versatility [11]. Recently, a variety of luminescent materials (e.g., organic fluorophores, luminescent inorganic nanocrystals, etc.) have been extensively studied, a number of which have limitations one needs to be aware of. For example, organic fluorophores suffer from rapid photobleaching and poor photostability [112]; and many nanosized luminescent materials have either colloidal stability or serious toxicity limits [113]. The encapsulation of such luminescent materials into the MSN scaffold provides a solution to tackle these aforementioned limitations. Moreover, due to both a high loading capacity and facile chemical tunability of the porous architecture, the use of MSNs can bring, as indicated above, much additional functionality. In this section, two typical MSN-based nanostructures that have been widely studied for optical imaging will be discussed (Figure 8).

### 4.1. Luminescent MSNs

#### 4.1.1. Organic Fluorophore-Incorporated MSNs

Organic fluorophores constitute a popular means of promoting optical imaging with molecular dyes, fluorescent proteins and fluorescent organic polymers commonly employed [114]. There exist a wide variety of OI-active organic moieties that have been incorporated into MSN-based nanomaterials, producing derived multifunctional agents. The introduction of fluorophores within MSN systems is achieved through covalent conjugation/physical adsorption either within the mesoporous channels or on the external surface of the nanoparticle [35]. The former can provide high photostability (the silica matrix prevents exposure of the organic dyes to the surroundings), while the latter can be used for a specially designed purpose such as gated drug delivery [115,116,117]. 

Co-condensation is a convenient way to introduce fluorescent functionality. For example, Lu et al. synthesised fluorescein isothiocyanate (FITC)-incorporated fluorescent mesoporous silica nanoparticles (FMSNs) by the co-condensation of an FITC-modified aminosilane [118]. The as-prepared FMSNs were injected in mice to investigate the toxicity and biodistribution of the particles. The accumulation of both FMSNs and folic-acid-conjugated FMSNs (F-FMSNs) were measured in human breast cancer cells (MCF–7), with the therapeutic ability of the loaded drug, camptothecin, on various cell lines (SK–BR–3, MCF–7 and MCF10F). The fluorescent active MSNs exhibited selective accumulation in tumour cells with promising levels of tumour suppression. Similarly, Huang et al. used fluorescence imaging to investigate the effects of MSN shape on biodistribution, clearance and biocompatibility in vivo [119]. In this study, FITC-functionalised FMSNs were synthesised with different reagent concentrations to achieve control over shape (producing short and long rod-like nanoparticles). It was found that the four types of FMSNs (both native and PEGylated short and long rod-like MSNs) were taken up by the reticuloendothelial system (RES) and observed to accumulate in different organs (e.g., lung, liver and spleen), exhibiting weaker fluorescent signals after seven days due to the metabolism of the nanoparticles. It was also concluded that both naked FMSNs and PEG-FMSNs had good biocompatibility and all exhibited low cytotoxicity in vivo. By using the co-condensation method, it is possible to evenly distribute organic dyes throughout the pore channels, with the outer silica framework forming a protective barrier that results in a greater dye photostability.

Post-grafting is an alternative method to incorporate organic dyes within the MSN scaffold and, in many cases, this has been combined with drug delivery. For example, Xie et al. reported a multifunctional MSN bioimaging probe both in vitro and in vivo, with anticancer drug (DOX) delivery in vitro [120]. MSNs with surface carboxyl functional groups were first synthesised, to which a Cy5 dye was then anchored forming fluorescent MSNs. The FMSNs were decorated with PEGylated folic acid (M_W_ ≈ 2000) and loaded with DOX. The as-synthesised nanoparticles exhibited low native cytotoxicity and a high intracellular uptake, with DOX release subsequently killing cancerous cells in vitro. Heidegger et al. reported an immune-responsive MSN composed of the pH-responsive poly(2-vinylpyridine) gatekeeper, an immune activator (TLR7 agonist R848) and fluorescent labelling with FITC [121]. In this work, there was a high uptake of FMSNs in antigen-presenting cells as resolved by confocal laser scanning microscopy, with a significant immune response (activation of lymphocytes) observed for these particles compared to cargo-free MSNs. Fan et al. have also developed luminescent HMSNs with drug delivery capabilities where amine-containing HMSNs were modified with an aggregation-induced emissive luminogen (BTPE) by post-grafting, with the particles subsequently loaded with DOX [122]. These nanoparticles afforded efficient intracellular imaging (bright blue emission). 

**Figure 9:**
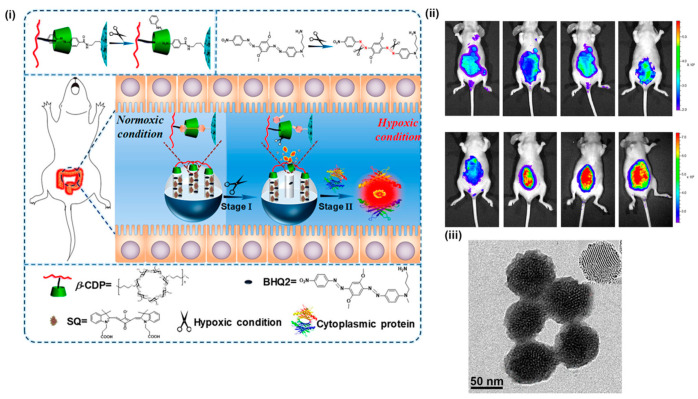
HCFA-MSNs. (**i**) A schematic illustration of hypoxia-activatable and cytoplasmic protein-powered fluorescence cascade amplifying (HCFA) MSNs. The HCFA-MSNs are composed of the β-cyclodextrin polymer (β-CDP) gatekeeper, loaded with both squarylium (SQ) dyes and black hole quencher 2 (BHQ2). (**ii**) In vivo fluorescence images of nude mice after the injection of a commercial small molecular fluorescent probe, the HCFA-MSNs, the control group or convalescent group of the HCFA-MSNs (from right to left), respectively. The most intense fluorescent signal can be observed the HCFA-MSNs. (**iii**) TEM image of the as-synthesised MSNs. Adapted with permission from Ref. [124]. Copyright (2020) American Chemical Society.

Aside from the covalent attachment of fluorescence-active moieties, there exist numerous examples where the organic fluorophore is physically entrapped within the pores of MSNs with Sreejith et al. reporting graphene-oxide-sheet-wrapped MSNs containing encapsulated squaraine dyes [123]. Here, graphene oxide (GO) sheets were electrostatically loaded on amino-functionalised MSNs, with the GO-wrapped nanocomposite providing a stable vessel for the squaraine dyes which exhibited a high chemical resistance to glutathione and cysteine. Similarly, in recent work reported by Zhou et al., squarylium (SQ) dyes were physically loaded into hypoxia-activatable and cytoplasmic protein-powered fluorescence amplifying (HCFA) MSNs [124]. In the HCFA-MSNs, a β-cyclodextrin polymer (β-CDP) was attached to the MSN surface, acting as a gatekeeper, and a black hole quencher 2 (BHQ2) was coloaded in the mesoporous channels capable of quenching SQ dyes. The injection of the as-prepared nanoparticles into mice with hypoxia-associated inflammatory bowel disease resulted in the removal of the β-CDP gatekeeper (under hypoxic conditions), releasing SQ dyes which coupled with cytoplasmic protein to amplify fluorescence intensity in vivo (Figure 9i–iii).

**Figure 10:**
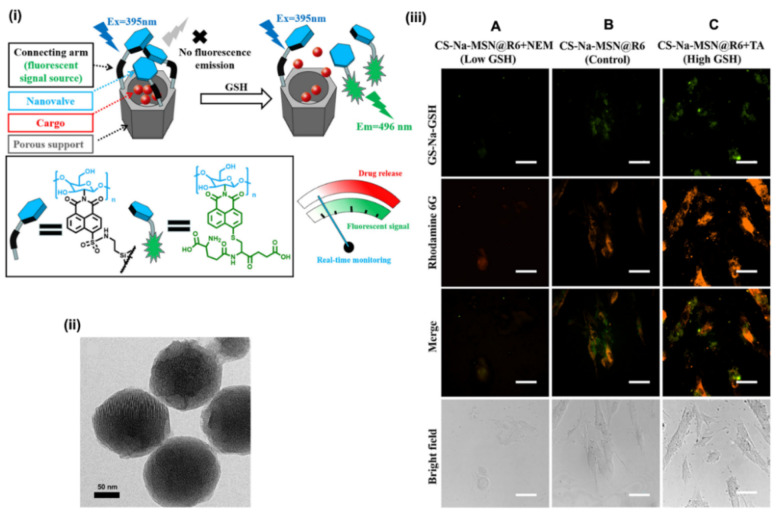
Chitosan-gated fluorescent mesoporous silica nanoparticles (FMSNs). (**i**) Schematic illustration of the design of chitosan-gated MSNs for monitoring drug release in vitro. The as-synthesised nanoparticles are composed of naphthalimide-dye-functionalised chitosan (Cs-Nac) as the gatekeeper, an MSN nanoparticulate scaffold and Rhodamine 6G (R6) as the model cargo agent. On glutathione (GSH) addition two fluorescent emissions corresponding to the release of R6 and cleaved Cs-Nac are observed. (**ii**) A TEM image of as-synthesised MSNs. (**iii**) Fluorescence images of MRC–5 cells with different GSH concentration. (A) The as-synthesised MSNs with low GSH concentration. (B) MSNs in MRC-5 without any other treatment. (C) MSNs with high GSH concentration. Adapted with permission from reference [126]. Copyright (2020) American Chemical Society.

In addition to pore-channel functionalisation, organic fluorophores can also be coupled to the exterior of MSNs, additionally acting as a gatekeeper. For example, Yang et al. developed fluorescent protein-tagged magnetic MSNs (M-MSNs) for drug delivery and fluorescence imaging [125]. The functional M-MSNs were composed of core/shell IONP/MS nanocomposites, His-tagged cyan fluorescent protein (HCFP) to act as a “nanovalve” and loaded with drugs (including DOX and IBU). The HCFP gatekeeper was removed on the addition of histidine, releasing the enclosed therapeutics, providing a promising platform to track drug release in vitro. Recent work by Chen et al. reported the use of chitosan-gated MSNs to monitor drug release in human lung fibroblast cells (MRC–5) [126]. A naphthalimide-dye-functionalised chitosan (Cs-Nac) was first synthesised and tethered to Rhodamine 6G (R6) loaded NH_2_-MSNs to form the gated nanosystem (Figure 10i–iii).

It was observed that, as the concentration of GSH increased, the electron-deficient sulfone from naphthalimide was cleaved from the MSNs through the sulfhydryl of GSH. As a result, increasing emission correlated with GSH concentration. The two emission enhancements were from (i) released R6 to give an intensity increase at 526 nm and (ii) cleaved chitosan groups which coupled with GSH to produce a new emission (λ_ex_ = 496 nm) through facilitated intramolecular charge transfer. This configuration, like others mentioned, could be readily applied to the direct imaging of therapeutic release.

#### 4.1.2. Luminescent Inorganic-Composite MSNs

In addition to organic dyes, there exist a broad range of luminescent inorganic materials (e.g., upconverting nanoparticles, quantum dots, carbon dots, photoluminescent metal complexes, etc.) that have been widely applied in bioimaging [127,128]. In recent years, for example, the use of persistent luminescent nanoparticles (PLNPs) for biomedical applications has attracted growing interest due to their long-lasting afterglow luminescence, facilitating imaging without continuous external illumination [128]. The default limitations of solubility and toxicity common to many of these can be alleviated through the incorporation within the MSN architecture. 

Carbon dots (CDs) are nanoscale carbon particles with a high photoluminescence performance, and have been incorporated within MSNs [129]. Chen et al. have, for example, reported a calcination (at 400 °C) method to convert amine-functionalised MSNs into fluorescent CD-containing MSNs [130]. The as-prepared nanoparticles showed strong fluorescence both in vitro and in vivo. Similarly, Kang et al. fabricated carbon-dot-incorporated HMSNs while also loading the nanocomposite with the anticancer drug DOX [131]. Carbon-dot-incorporated MSNs (CD-MSNs) can also be fabricated by traditional sol–gel reactions employing a carbon dot precursor. This has been reported by Lei et al. where CD-PMO nanoparticles were synthesised by a co-condensation reaction using TEOS, CTAB and ultra-small organosilane-functionalised CDs [132]. The as-prepared CD-PMO nanoparticles have been shown to exhibit two-photon fluorescence in cells, with excellent tunability of this by altering the reactant ratio.

In addition to carbon dots, lanthanide (e.g., europium, terbium, ruthenium, etc.) complexes have been covalently tethered to the MSN framework. This has been shown, for example, by Liu et al. where lanthanide-incorporated MSNs were able to perform in vitro luminescence imaging, exhibiting multi-colour luminescence ranging from visible to near infrared [133]. Alternatively, transition metal complexes have been incorporated within MSNs. For example, Wang et al. developed fluorescence resonance energy transfer (FRET)-based MSNs with covalently incorporated zinc 8-hydroxyquinolinate (Znq) inside the mesopores and PEG-folate anchored to the external surface [134]. When DOX was loaded into the nanosystem, the nanoparticles exhibited a strong FRET effect (from FMSNs to DOX) which significantly decreased the green-colour emission of the FMSNs. FRET quenching was reduced on DOX release, enabling a useful direct imaging of this. Work by Kitajima et al. reported the internal coupling of the phosphorescent transition metal complex bis(2,2′-bipyridine)-(5-maleimide-1,10-phenanthroline) ruthenium(II) within the pores channel of the MSNs for oxygen sensing both in vitro and in vivo [135]. Compared to direct excitation of the molecular ruthenium analogue, these nanoparticles demonstrated significant reduced levels of singlet oxygen (^1^O_2_) generation, reducing cytotoxicity and providing the potential to probe oxygen levels in vitro and in vivo.

**Figure 11:**
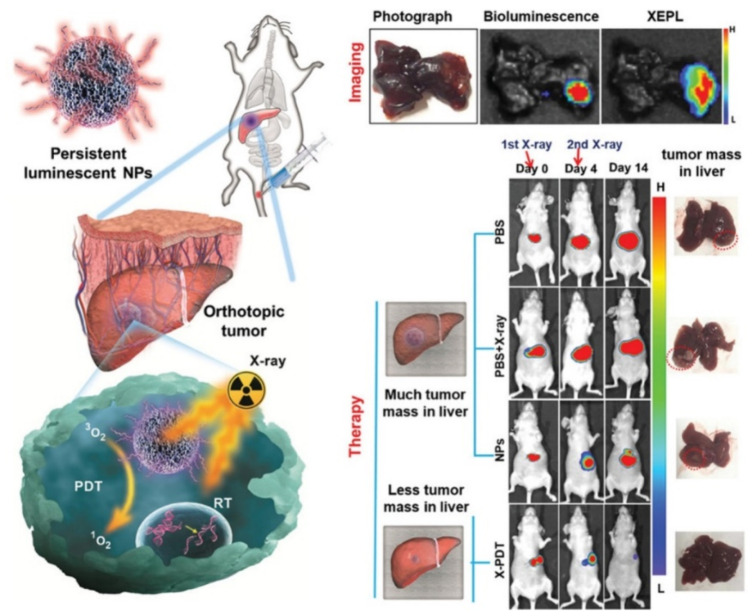
Schematic illustration of persistent luminescent nanocrystal-incorporated MSNs for afterglow luminescence imaging and photodynamic therapy in vivo. The as-synthesised nanoparticles are composed of the mesoporous silica-templated zinc gallogermanate (mZGGOs) nanocomposite core, an outer PEGylated coating and the loading of silicon phthalocyanine photosensitiser (Si-Pc). This design exhibited rechargeable X-ray-excited persistent luminescence (XEPL) properties for bioimaging and the ability of tumour suppression. Reprinted with permission from reference [139]. Copyright (2020) Wiley-VCH.

Persistent luminescent nanoparticles (PLNPs) can be synthesised by various methods including both sol–gel and template chemistry, with MSNs, as of writing this review, the only successful template offering control over morphology [136]. MSN templates can, for example, be used to generate multifunctional MSN/PLNP nanocomposites with Li et al. reporting a facile fabrication of persistent luminescent SrMgSiO_6_:Eu^2+^_0.01_,Dy^3+^_0.02_ nanocomposites by a high-temperature conversion of the metal (Sr^2+^, Mg^2+^, Eu^2+^, Dy^3+^) impregnated MCM-41 [137]. The nanocomposites were then grafted with PEG groups, exhibiting long afterglow phosphorescence. Three years later, Li et al. published near infrared (NIR)-emitting MSN/PLNP for persistent luminescence imaging in vivo [138]. This nanoparticulate example was capable of rechargeability (repeated activation) of the afterglow luminescence in vivo. Work by Shi et al. reported X-ray-excited persistent luminescent (XEPL) particles for both bioimaging and photodynamic therapy [139]. Here, XEPL nanoparticles were synthesised by the high-temperature (850 °C) calcination of the metal (Zn, Ga, Ge, Cr^3+^, Yb^3+^ and Er^3+^) loaded MSNs, followed by surface grafting of PEG and photosensitiser (silicon phthalocyanine, abbreviated to Si-Pc) loading. Under X-ray irradiation, this configuration was able to emit long-lasting afterglow luminescence in the NIR region and generate singlet oxygen to inhibit tumour growth (Figure 11).

### 4.2. Luminescent Core–Shell MSNs

Core–shell structured MSNs with a luminescent inorganic core and mesoporous silica shell present another potent option for optical imaging. Distinct from nanocrystal-incorporated MSNs (Section 4.1.2), the desired core–shell structure is often built from the preformed inorganic nanocore, with a subsequent sol–gel growth of mesoporous silica [140]. Some common inorganic luminescent core materials include upconverting nanoparticles (UCNPs), persistent luminescence nanoparticles (PLNPs), etc. [11,141,142]. 

Recent advances in the core/shell UCNP/MSN hybrid materials include the development of a triggerable core–shell UCNP@MSN nanocomposite with an azobenzene-modified silica shell, loaded with the anticancer drug DOX [143] by Liu et al. Upon NIR irradiation, the UCNP core emits UV/visible light, and triggers a reversible isomerisation of the azo groups within the mesoporous silica shell. This photo-isomerisation apparently encourages the release of the loaded DOX. Lai et al. reported mesoporous-silica-coated UCNPs with both interior DOX loading and exterior ATP responsivity, and employed for real-time monitoring of drug delivery in cells [144]. 

**Figure 12:**
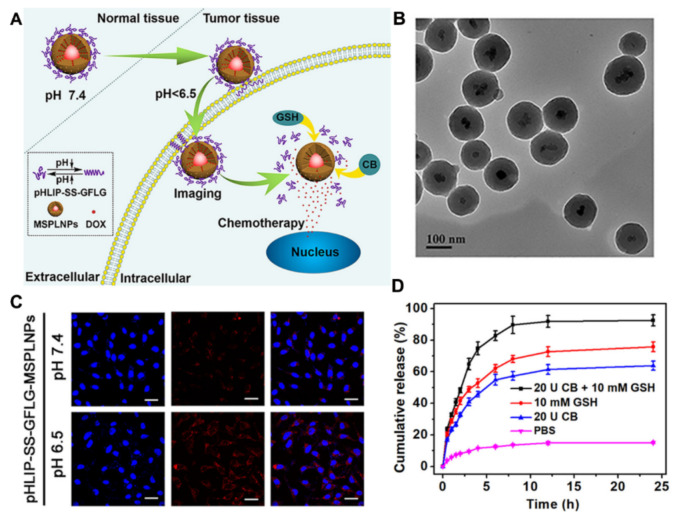
(**A**) Schematic illustration of DOX-loaded zinc gallogermanate (ZGGO)@mSiO_2_@GFLG-SS-pHLIP composed of persistent luminescent nanoparticle (PLNP)/MSN core/shell structure, glutathione/cathepsin B dual-responsive polypeptide coating (pHLIP-SS-GFLG) and anticancer drug (DOX). This nanosystem is designed to achieve drug delivery under glutathione or/and cathepsin B conditions. (**B**) TEM image of ZGGO@mSiO_2_. (**C**) Confocal laser scanning microscopy images of stained A549 cells, ZGGO@mSiO_2_@GFLG-SS-pHLIP, and merged (from left to right). (**D**) The drug release profile of DOX-loaded ZGGO@mSiO_2_@GFLG-SS-pHLIP in PBS buffer solution with or without stimulus (glutathione and cathepsin B). Adapted with permission from reference [147]. Copyright (2020) American Chemical Society.

Core/shell PLNP/MSNs have also been of recent interest, particularly the use of PLNPs in the near infrared range (e.g., zinc gallogermanate (ZGGO)). The imaging window of near infrared enables a deeper tissue penetration, reducing the potential photocytotoxicity. For example, Liu et al. reported the synthesis of core/shell ZGGO/MSNs coated with erythrocyte membrane for drug delivery in vivo [145]. Similarly, Wang et al. have developed core/shell ZGGO@mesoporous silica shell (mSiO_2_) nanoparticles with a tumour targeting biofilm coating, capable of targeted drug delivery, tracked by luminescent imaging in vivo [146]. Recent work by Zhang et al. developed a ZGGO@mSiO_2_-based acid-driven nanoprobe with dual-responsive drug release (Figure 12) [147].

In this example, ZGGO@mSiO_2_ nanoparticles were coated with a polypeptide which is composed of a part of a pH-low-insertion peptide (pHLIP) and a part of a GFLG peptide with the linkage of a disulphide bond (pHLIP-SS-GFLG). This system was subsequently loaded with anticancer drug DOX. The particles were observed to exhibit the strongest cellular (A549 cells and HepG2 cells) luminescence intensity under acidic conditions (pH 6.5) compared to physiological pH (pH 7.4), an observation assigned to a triggered conformational change in the peptide, facilitating its membrane insertion. At the same time, in vitro drug release was demonstrated in a buffer solution containing glutathione (GSH) and/or cathepsin B (CB), with the highest release observed when both were present.

## 5. Other Imaging Modes and Multi-Modal Imaging

### 5.1. Other Imaging Modalities

#### 5.1.1. Positron Emission Tomography

Positron emission tomography (PET) has become a popular imaging technique due to its non-invasive nature and high sensitivity [148]. PET imaging employs a positron-emitting radioisotope such as fluorine-18 (^18^F), copper-64 (^64^Cu) or zirconium-89 (^89^Zr), with the emitted photon detected by a germanium-based detector. It is worth noting that the different radioisotope half-lives (*T*_1/2_), e.g., ^18^F: 109.7 min, ^64^Cu: 12.7 h, ^89^Zr: 78.4 h, etc., constitute an important practical consideration. 

Recently, MSN-based PET contrast agents labelled by radionuclides with a long half-life have been investigated. For example, Ellison et al. reported radioarsenic MSNs for in vivo imaging [149]. This was achieved by the coupling of arsenic trioxide (ATO) with the PET-active isotopes ^72^As and ^74^As (both have *T*_1/2_ greater than a day) through thiol groups present on the MSN surface. Goel et al. reported biodegradable mesoporous silica nanoparticles (bMSNs) with ^89^Zr radiolabelling (*T*_1/2_ = 78.4 h) for an in vivo pharmacokinetic study (complete degradation seen within 21 days) [150]. In this case, a cancer-specific antibody (TRC105) was conjugated onto the surface of bMSNs with the hydrophobic anti-angiogenesis drug (sunitinib malate) loaded into the mesopores of the MSNs, providing excellent tumour-cell (CD105) specificity in combination with effective controlled release. Similarly, recent work by Xu et al. described multifunctional bMSNs radiolabelled with ^64^Cu (*T*_1/2_ = 12.7 h) for cancer imaging and photodynamic therapy [151]. 

**Figure 13:**
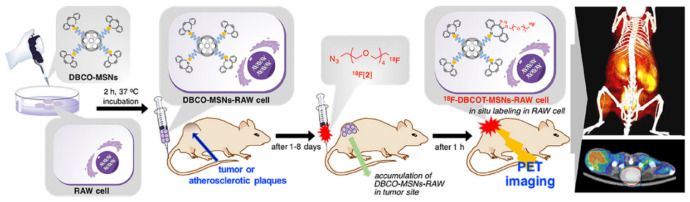
A schematic representation for the design of ^18^F-radiolabelled MSNs. Cyclooctyne functionalised MSNs (DBCO-MSNs) were first incubated with RAW cells, with the incubated samples then injected into nude mice bearing a tumour or atherosclerosis plaques. One to eight days later, ^18^F-labelled azide was injected into the mice to visualise the tumour/atherosclerotic sites in vivo. Reprinted with permission from reference [152]. Copyright (2019) Elsevier.

Radionuclides with short half-lives have also been incorporated into well-defined mesoporous silica nanomaterials through efficient conjugating/loading. For example, recently, Jeong et al. described ^18^F (*T*_1/2_ = 109.8 min)-labelled MSNs for in vivo imaging, with conjugation achieved by a strain-promoted alkyne azide cycloaddition (SPAAC) (Figure 13) [152].

More specifically, a PEGylated MSNs surface was functionalised with cyclooctyne, generated and intravenously injected into the mouse, with the ^18^F-labelled azide species then injected to couple with the functionalised MSNs a few days later. With the help of the SPAAC reaction, the accumulation of radiolabelled nanoparticles in vivo was shown, with tumour and atherosclerotic sites thereby visualised through PET imaging. More recently, ^68^Ga (*T*_1/2_ = 68 min)-labelled MSNs have been reported by Jung et al. to serve as a PET imaging probe [153]. Radioactive ^68^Ga was physically loaded within the silica matrix by a chelator-free labelling method, enabling the conjugation of the radiotracer to the MSNs in less than 20 min.

#### 5.1.2. Computed Tomography

CT imaging constitutes three-dimensional anatomical imaging based on differences in X-ray attenuation, with an associated high spatial resolution and low cost. It is, then, a highly important modality in diagnostics [154]. Current clinical contrast agents for CT imaging are based on iodine analogues which suffer from poor blood circulation times, in addition to potential renal toxicity and anaphylaxis [155]. Encapsulation within a nanoparticulate structure can improve both biocompatibility and retention times, facilitating the use of CT agents such as gold and bismuth that have improved X-ray attenuation over iodine analogues, and thus sensitivity, permitting a lower concentration of the contrast agent to be employed in vivo [156]. The MSN scaffold allows for facile tethering of chemical moieties in addition to the ability to act as a drug reservoir. Baek et al. have reported the use of Au nanorods encapsulated within an MS shell for CT imaging and DOX loading [157]. The nanoparticles were capped with the thermo-responsive polymer poly(*N*-isopropylacrylamide-*co*-*N*-butylimidazolium) (poly(NIPAAm-*co*-BVIm) to allow for NIR-triggered drug delivery. Au NPs have the ability to generate heat from an NIR light source (λ = 600–900 nm) through localised surface plasmon resonance (SPR) properties allowing additional PTT. As such, irradiation of the nanoparticles with NIR caused an increase in temperature to 43 °C, releasing the loaded DOX. Au NPs have also been coated in a mesoporous silica shell through a sol–gel method to produce Janus-type nanoparticles [158]. CT imaging was facilitated through the Au core allowing for the identification of hepatocellular carcinoma in vivo, with folic acid conjugation to the MS shell for active targeting. The SPR properties of the Au core enabled radiotherapy through PTT, producing a versatile theragnostic platform. Bimetallic nanoparticle systems have also been reported to enhance CT attenuation, with tantalum oxide core/mesoporous shell nanoparticles decorated with Au NPs on the surface reported by Kashfi-Sadabad et al. [159]. Both gold and tantalum oxide have high attenuation coefficients making them effective contrast agents for CT imaging. Bismuth sulphide nanoparticles are also commonly employed to enhance CT image contrast with the high X-ray attenuation of Bi due to its high atomic number. The narrow bandgap of Bi_2_S_3_ NPs absorbing in the NIR region also facilitates hyperthermia in tumour cells promoting cell death. Lu et al. have coated Bi_2_S_3_ NPs with an MS shell to encapsulate DOX and allow for NIR-triggered DOX release and PTT, in addition to CT image enhancement, with the silica shell functionalised with cRDG (arginine-glycine-aspartic acid) for active tumour targeting through α_v_β_3_ and α_v_β_5_ integrin receptors [160]. 

### 5.2. Multi-Modal Imaging

Associated with each individual imaging modality is a set of unique advantages and limitations; here is born the advantages of a multi-modal agent. For example, magnetic resonance imaging (MRI) has associated high spatial resolution but low sensitivity with OI being orders of magnitude more sensitive but with low penetration depth. Mesoporous silica has been widely used as a scaffold for the construction of multifunctional nanoprobes due to the accessible rich synthetic manipulation discussed above. Early MSN-based works on multimodality in imaging have been previously reviewed [10,161,162]. This section highlights more recent examples, subdividing architectures into those based on the functionalisation of MSNs with paramagnetic/radioactive/optical moieties (Section 5.2.1) and core–shell structured MSNs (Section 5.2.2).

#### 5.2.1. Functional Moiety Incorporated MSNs

As noted, the facile chemical tunability of the MSN architecture allows the incorporation of additional imaging modalities through abundant silanol groups present on the surface/within the pore channels. Sun et al. have, for example, reported the conjugation of indocyanine green-loaded thermosensitive liposomes to Gd-doped and DOX-loaded MSNs for dual-modal MRI and fluorescence imaging. The thermosensitive liposomes were further modified with FA for active targeting of cancerous tissue. Under near infrared radiation, indocyanine green generates heat resulting in the cleavage of the liposomes and release of the loaded therapeutic DOX [163]. Dual-modal MRI and fluorescence imaging has also been reported by Carniato et al. where Gd-DOTA-functionalised MSNs were additionally tethered with a fluorescent active rhodamine dye. These particles were loaded with the chemotherapeutic mitoxantrone, with the Gd-chelates promoting MRI activity with associated *r*_1_ = 49.2 mM^−1^ s^−1^ at 0.47 T [164]. Further dual-modal optical/MRI-active MSN examples include the doping of ZnSe QDs with manganese and loading within the silica mesopores, with Mn providing MRI contrast capabilities and the QD core fluorescence [165].

Dual-modal SPECT/MRI agents have also been reported; e.g., Gao et al. produced ^99m^Tc-conjugated manganese-oxide-loaded MSNs for SPECT imaging and pH-responsive MRI. The manganese oxide nanoparticles loaded within the pores of the MSNs disassemble at low pH, releasing MRI-active Mn^2+^ with an increase in longitudinal relaxivity from *r*_1_ = 0.58 mM^−1^ s^−1^ to *r*_1_ = 6.60 mM^−1^ s^−1^. The labelling of the MSN surface with the radionuclide ^99m^Tc enabled SPECT imaging, producing, then, a nanoparticulate platform with both high spatial resolution and sensitivity [166]. 

#### 5.2.2. Core–Shell Structured MSNs

**Figure 14:**
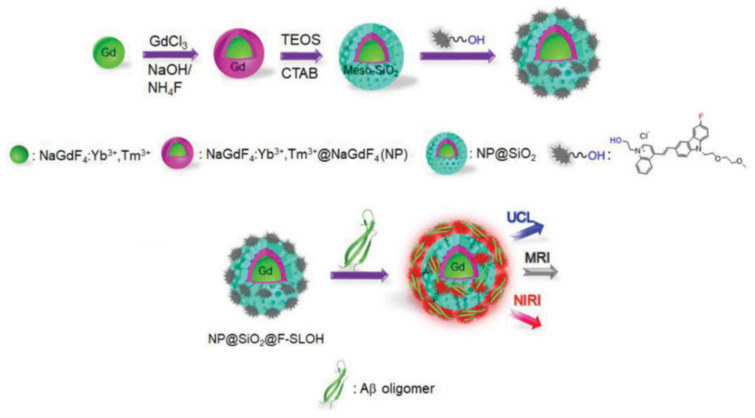
Schematic illustration of mesoporous silica-coated upconversion nanoparticles composed of a gadolinium doped UCNP core (noted as NP), mesoporous silica shell (labelled SiO_2_) and Aβ oligomer-selective cyanine dye coating (noted as F-SLOH). The gadolinium-doped UCNP core provides *T_1_* MRI contrast and the selective binding of Aβ oligomers to the F-SLOH dye facilitates target activated NIR fluorescence (denoted near infrared imaging, NIRI). UCL refers to upconversion luminescence. Reprinted with permission from reference [171]. Copyright (2020) Wiley-VCH.

Lanthanide-based core–shell structured mesoporous silica nanoparticles have been widely used for multi-modal probes capable of biomedical imaging (e.g., early diagnosis of cancer) [167]. For example, lanthanide-doped upconversion nanoparticles (UCNPs) coated in mesoporous silica are not only able to facilitate upconversion luminescence (UCL) imaging or near infrared fluorescent imaging [168], but also the enhancement of MR contrast especially through the use of dopants such as gadolinium [169]. This is exemplified in, for example, work by Qiao et al., where mesoporous silica-coated gadolinium-doped upconversion nanoparticles for MR/UCL have been reported for both dual-modal imaging and osteocyte-targeting therapy [170]. Another recent example by Wang et al. where mesoporous silica-coated UCNPs additionally coated with a biomarker-selective fluorophore were used for both identification and therapy of Alzheimer’s disease (Figure 14) [171].

Such formulations have also been used for the sensing of Hg^2+^, where Ge et al. used UCNPs coated in an MSN shell and tethered to a Ru chromophore. Here, the UCNPs are energy donating to the Ru complex, with this energy transfer inhibited on the addition of Hg^2+^, resulting in an increase in green emission intensity. The surfaces of the particles were functionalised with polyethyleneimine (PEI) to improve biocompatibility, and doping of the UCNP core with Gd^3+^-facilitated additional MRI activity [172]. Different core doping can also introduce alternative imaging capabilities as reported by Lv et al. where the introduction of Yb to the UCNP core allowed CT imaging [173]. Gadolinium (III) oxide nanoparticles can also be encapsulated in mesoporous silica to introduce image contrast within multimodal agents [174]. 

**Figure 15:**
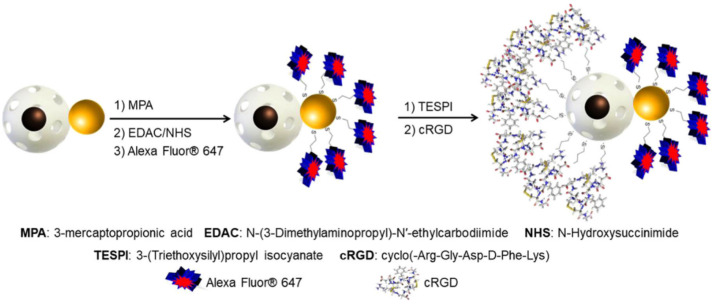
A schematic representing a multi-modal Janus nanoparticulate system composed of a SPION/MSN core/shell face and Au nanoparticle face. *T*_2_ MRI contrast capabilities arise from the encapsulated SPION with Au nanoparticles providing the possibility for CT imaging. As shown in this schematic, the system is functionalised with a fluorescent dye (Alexa Fluor^®^ 647) for optical imaging in addition to cRDG for active tumour targeting. Reprinted with permission from reference [178]. Copyright (2018) American Chemical Society.

SPION core/MSN shell nanocomposites are an alternative choice as multi-modal MSN-based imaging systems and such adducts have been reported with additional OI capabilities by grafting CdSe/ZnS QDs, which have an emissive wavelength of 630 nm, within the silica pore channels [175]. These particles were also functionalised with HSA to improve biocompatibility and blood circulation time. Computed tomography (CT) has often been combined with MRI, where its associated high sensitivity produces combined contrast agents with both high spatial resolution and unparalleled sensitivity [73]. In work by Tseng et al. this has also been complemented by additional tumour targeting abilities and pH/*cis*-diol responsive controlled drug release [176]. Here, the MSN core with SPIONs adsorbed onto the surface was encapsulated within a thin mesoporous shell. CT-active, Au nanoparticles were tethered to act as a pH-sensitive gatekeeper through hydrolysable boronate ester bonds for DOX release within an acidic microenvironment. Folic acid was also linked to the Au nanoparticles for active tumour targeting. Janus-type nanoparticles, where the system is sphero-ellipsoidal in shape, constitute potentially interesting multi-modal platforms [177]. SPION-encapsulated MSNs have been used to present one face, with an Au nanoparticle as the other face in hybrid system supporting MRI and CT image contrast [178]. The gold interface was modified with a fluorescent dye (Alexa Fluor^®^ 647) and the MSN face with a tumour-targeting peptide cRDG (Figure 15). 

In addition to UCNP and SPION nanocores, other functional nanoparticles have also been encapsulated within the mesoporous silica matrix in supporting multimodality. For example, Song et al. reported gold nanosphere core/MSN shell nanoparticles for dual-modal CT/fluorescence imaging in vivo [179]. In this work, Au NPs formed the CT active core, with NIR dyes (IR–783) electrostatically loaded within the mesoporous silica shell to act as a fluorescence imaging probe. Similarly, Chakravarty et al. have reported the use of tantalum oxide (TaO_x_) as a CT active core with FITC, a fluorescent active dye, conjugated to the MS shell [180]. 

## 6. Conclusions and Future Outlook

Mesoporous silica nanoparticles have wide ranging applicability in medical imaging, owing to high chemical tunability, control over morphology and the possibility of additional drug or nanoparticle loading. The versatility of this platform is undoubtedly the driving force behind its ever-increasing application as a bioimaging platform, with real potential to support the diagnosis of a plethora of diseases in vivo. The chemical richness of MSN adducts has been exemplified in multimodal imaging, specific targeted drug delivery, stimuli-responsivity and in phototherapy applications. Although bioaccumulation studies are complex and must necessarily continue, it is clear that biocompatibility is good [181,182,183,184,185] and can be further tuned through, for example, passivating polymer coatings. Effective generated image contrast has been observed and exemplified through the examples included in this review offering improved diagnostic potency over corresponding molecular analogues. The ability to localise paramagnetic chelates within the pore channels markedly improves MRI contrast, through greater relaxivities, arising from the geometric confinement effect. An additional loading with OI agents within the pores/core of MSNs is associated with an improved probe thermo- and photo-stability. The ease of synthesis and richness of functionality is such that the lessons learnt thus far with MSNs are likely to underpin much development in theragnostics over the next decade.

## Figures and Tables

**Table 1:** Common types of mesoporous silica nanoparticles (MSNs).

MSNs Type	Morphological Structure	Pore Size (nm)	Surfactant in Use	References
MCM-41	2D hexagonal *p6mm*	1.5–8	C_n_TMA^+^ (n = 12–18) ^a^	[19,20,21]
MCM-48	3D cubic *Ia3d*	2–5	C_n_TMA^+^ (n = 16) C_16-12-16_	[22,23]
MCM-50	Lamellar *p2*	2–5	C_n_TMA^+^ (n = 16)	[19,24]
SBA-3	2D hexagonal *p6mm*	2–4	C_n_TMA^+^ (n = 14–18)	[19,25]
SBA-15	2D hexagonal *p6mm*	5–10	Gemini surfactants ^b^	[26,27,28]
KIT-5	Cubic *Fm3m*	∼9.3	F–127 ^c^	[29,30]
FDU-12	Cubic *Fm3m*	10–27	F–127	[31,32]

^a^ Alkyltrimethylammonium salts; ^b^ Some examples such as P123, P85, P65, Brij 97; ^c^ Pluronic triblock polymer; MCM—Mobil Composition of Matter; SBA—Santa Barbara Amorphous; KIT—Korea Advanced Institute of Science and Technology; FDU—Fudan University.
